# Birth and death evolution of *polyphenol oxidase* (*PPO*) gene family in *Oryza* species

**DOI:** 10.1186/s40529-026-00491-5

**Published:** 2026-02-05

**Authors:** Yuan-Ching Tsai, Yi-Jing Chen, Sou-Yu Cheng, Charng-Pei Li, Ming-Der Huang

**Affiliations:** 1 Department of Agronomy, National Chiayi University, Chiayi City, Taiwan; 2 Department of Biological Sciences, National Sun Yat-Sen University, Kaohsiung, 80424 Taiwan; 3 Taiwan Agricultural Research Institute (TARI), Ministry of Agriculture, Taichung City, Taiwan

**Keywords:** Polyphenol oxidases, Rice, *Oryza* species, Gene cluster, Truncated PPOs, Enzymatic browning

## Abstract

**Background:**

Polyphenol oxidases (PPOs) are copper-containing enzymes that catalyze the oxidation of phenolic substrates to quinones. In cereal crops, PPOs contribute to both grain browning and defense responses. During domestication, multiple independent *PPO* mutations have been selected, creating a trade-off between grain quality and plant fitness. In rice, most PPO-related studies have focused on the reference genome of *Oryza sativa* ssp. *japonica* cv. Nipponbare, yet a comprehensive understanding of *PPO* gene diversity, copy number variation, and evolutionary history across the *Oryza* genus remains lacking.

**Results:**

We systematically identified *PPO* genes in 21 fully sequenced wild and cultivated *Oryza* species, along with three close relatives. PPOs were classified into three types (PPO1–3), with PPO1 further divided into two subtypes (PPO1-1 and PPO1-2) and PPO3 is reported here for the first time. Both PPO1 and PPO3 possess thylakoid transfer domains and Tat-specific motifs, whereas PPO2 lacks these features. *PPO1* and *PPO2* expanded through tandem duplications, forming two major clusters: the *PPO1* cluster and the *PPO2*/*PPO3* cluster. Truncated PPO variants were found to be widespread across *Oryza*, present in roughly half of the surveyed species and often outnumbering intact PPO counterparts. Most truncated *PPO*s contain multiple indels: those in *PPO1-1* and *PPO3* arose independently, whereas the truncations in *PPO1-2* and *PPO2* are conserved. Furthermore, PPO mutations in Nipponbare (*PPO1* and *PPO3*) and *O. glaberrima* (*PPO1-1*) are associated with domestication, with *PPO1-2.1* in Nipponbare disrupted by transposable-element insertions and *O. glaberrima* harboring a novel truncated *PPO1-1* allele.

**Conclusions:**

This study provides the first comprehensive analysis of the *PPO* gene family across *Oryza*, revealing three major PPO types. The expansion of PPOs through tandem duplications, coupled with frequent loss-of-function mutations due to frameshift-indel truncations, is consistent with a birth-and-death model of gene family evolution. Our findings highlight the combined contributions of domestication and natural selection in shaping *PPO* diversity in *Oryza*.

**Supplementary Information:**

The online version contains supplementary material available at 10.1186/s40529-026-00491-5.

## Introduction

Polyphenol oxidases (PPOs) are copper-containing enzymes that catalyze the hydroxylation of monophenols and the oxidation of *o*-diphenols to *o*-quinones in the presence of oxygen (Tomás-Barberán and Espín 2001; Mayer 2006). The resulting reactive quinones undergo non-enzymatic polymerization with amino acids and proteins, forming brown to black melanin pigments (Mayer and Harel 1979). The most recognized manifestation of PPO activity is enzymatic browning in fruits and vegetables following mechanical injury, a process that can adversely affect food quality and consumer acceptance (Sapers and Miller 1993). Beyond postharvest physiology, PPOs are implicated in diverse physiological and metabolic processes, particularly in plant responses to tissue damage and biotic stress. Upon wounding or pathogen infection, PPO-mediated oxidation of phenolic substrates generates reactive *o*-quinones that participate in redox modulation, protein cross-linking, and the formation of antimicrobial or deterrent compounds (Constabel et al. 2008; Zhang 2023). Genetic and biochemical studies in multiple plant species have shown that elevated PPO activity correlates with increased resistance to herbivory, whereas PPO suppression is associated with enhanced susceptibility (Thipyapong et al. 2004; Mahanil et al. 2008). In wild oat and tobacco, pathogen infection has been proposed to trigger proteolytic activation and release of PPO, generating antimicrobial *o*-quinones and melanins that may contribute to the resistance response (Fuerst et al. 2014; Aziz et al. 2019). Furthermore, PPOs have been associated with plant development and specialized metabolism. In certain tissues, PPO activity is linked to phenylpropanoid metabolism, lignification processes, and pigmentation, suggesting broader metabolic functions beyond plant defense (Mayer 2006; Araji et al. 2014; Sullivan 2015).

Structurally, the biochemical activity and regulated deployment of plant PPOs are supported by a conserved multi-domain architecture. Plant PPOs typically consist of three domains: an N-terminal chloroplast targeting sequence, a dicopper catalytic tyrosinase domain, and a C-terminal regulatory region (Tran and Constabel 2011). The N-terminal region of canonical plant PPO contains both chloroplast transit peptide (cTP) and thylakoid transit domain (TTD). The cTP directs PPO import into the chloroplast stroma and then the TTD subsequently guides the PPO to thylakoid lumen via the twin-arginine translocation (Tat) pathway. Both cTP and TTD are subsequently cleaved by signal peptidase, leaving PPO in a latent state within chloroplasts (Koussevitzky et al. 2008). The catalytic core contains two conserved copper-binding sites, CuA and CuB, each coordinated by three histidine residues (Klabunde et al. 1998). CuA contributes to protein solubility, whereas CuB directly mediates substrate oxidation (Mayer 2006; Zhang 2023). The C-terminal domain (CTD), typically 15–20 kDa, functions as a latency-inducing shield that occludes the active site and prevents catalysis until proteolytic cleavage or stress exposure activates the enzyme (Robinson and Dry 1992). While the CTD exhibits sequence variation among plant PPOs, it contains conserved structural motifs such as DWL and KFDV that are thought to be important for regulatory function (Tran et al. 2012). Despite its role in PPO regulation, the CTD remains relatively understudied compared to the catalytic core, and the evolutionary dynamics and functional significance of CTD variation across plant lineages are poorly understood.

PPOs are widely distributed across microorganisms, animals, and plants, but are largely absent or rarely reported in green algae (Tran et al. 2012; Zhang 2023; Zou et al. 2025). In plants, PPOs are predominantly localized in plastids, including chloroplasts of photosynthetic tissues and leucoplasts of storage cells, whereas their phenolic substrates are typically sequestered in the vacuole (Tran and Constabel 2011; Zhang 2023). This spatial separation prevents premature oxidation under normal conditions. Upon tissue damage, this compartmentalization is disrupted, allowing PPO to interact with vacuolar phenolic substrates and oxygen, thereby initiating the enzymatic browning reactions (Vaughn and Duke 1984; Constabel et al. 1996). While most PPO isoforms reside in plastids, certain variants display alternative localizations. For instance, PtrPPO13 in poplar contains an N-terminal signal peptide that directs it to the vacuole, whereas *Physcomitrella* PPO1 has been reported to localize extracellularly, where it may contribute to the detoxification of phenolic compounds in the apoplast (Tran and Constabel 2011; Richter et al. 2012). These diverse subcellular localizations highlight functional specialization among PPO isoforms and suggest broader roles for PPOs beyond their canonical plastid-associated activity.

Plant PPOs are encoded by nuclear multigene families, with copy numbers varying widely across lineages (Newman et al. 1993; Zhang 2023). Non-vascular plants can harbor large PPO families, with 13 genes in *Physcomitrella patens* (Richter et al. 2012) and 34 in *Marchantia polymorpha* (Furudate et al. 2023). In contrast, vascular plants typically contain fewer *PPO* genes, ranging from two in *Oryza sativa* (Yu et al. 2008) to four in *Hordeum vulgare* (Glagoleva et al. 2024), seven in *Lycopersicon esculentum* (Newman et al. 1993), nine in *Solanum tuberosum* (Chi et al. 2014), 13 in *Nicotiana tabacum* (Zhang et al. 2025), 14 in *Populus trichocarpa* (He et al. 2021), and up to 26 in *Salvia miltiorrhiza* (Zhang et al. 2020). Moreover, *PPO* genes are completely absent from Arabidopsis and Brassica genomes (Tran et al. 2012). Collectively, these patterns suggest that PPO family expansion has not scaled consistently with plant evolutionary complexity (Tran et al. 2012).

Loss-of-function mutations in *PPO* genes have been reported in several domesticated crops, including foxtail millet (Inoue et al. 2015; Fukunaga et al. 2020), rice (Yu et al. 2008; Gross et al. 2009), barley (Taketa et al. 2010), and pea (Balarynová et al. 2022). These mutations often arise from single or multiple nucleotide substitutions or transposon insertions that introduce premature stop codons, leading to truncated PPO proteins (Gross et al. 2009). Such alleles are commonly associated with crop domestication, as reduced PPO activity diminishes enzymatic browning, and improves the appearance and palatability of food products (Chi et al. 2014). In rice, a loss-of-function allele of the major *PPO* gene *Phr1* is predominantly found in *japonica* cultivars, resulting in the characteristic negative phenol-staining phenotype. Population genetic analyses indicate that positive selection favored these loss-of-function alleles during *japonica* domestication. Conversely, functional *Phr1* alleles are maintained in *indica* subspecies through balancing selection, potentially reflecting agricultural pressures in tropical and subtropical climates that favor traits associated with disease resistance and seed dormancy (Yu et al. 2008). Similarly, weedy rice populations retain PPO activity through balancing selection, as seed dormancy represents a critical adaptive trait preventing premature germination under variable environmental conditions (Gross et al. 2009). Weedy rice shows low frequencies of phenol-negative variants (~3.7%), primarily originating from crop-to-weed gene flow rather than de novo mutations, suggesting that PPO activity is adaptive in weedy populations (Gross et al. 2009). Previous studies identified two full-length *PPO* loci in rice, *Phr1(PPO1*) and *Phr1L3*(*PPO2*), with *Phr1* being directly responsible for seed browning (Yu et al. 2008). However, the *japonica* reference genome (cultivar Nipponbare) also contains additional truncated *PPO* sequences, including mutated *Phr1* and two nearby loci (*Phr1L1* and *Phr1L2*), suggesting that the rice PPO family may be more complex than previously recognized and warranting comprehensive characterization across diverse rice germplasm.

In this study, we systematically identified and characterized *PPO* genes across 19 wild and two cultivated *Oryza* species to investigate their genomic distribution, structural variation, and evolutionary patterns. Through phylogenetic analysis and protein sequence alignment, we classified *Oryza PPO*s into three distinct types (PPO1–PPO3) and found that *PPO1* and *PPO2* have undergone lineage-specific expansion via tandem duplication, forming distinct gene clusters. Our analysis also revealed that truncated *PPO* variants are prevalent across most *Oryza* species, with genome-specific mutation patterns indicating that the *PPO* gene family has been shaped by both gene expansion and gene loss during rice evolution. Furthermore, we identified a novel loss-of-function allele of *PPO1* in the cultivated African rice *Oryza glaberrima* and confirmed its non-functional status through phenol staining assays. These findings provide insights into the evolutionary dynamics of the *PPO* gene family in rice and its relationship to domestication and natural selection.

## Materials and methods

### Identification of polyphenol oxidase (PPO) genes in rice genomes

Genomic databases of wild and cultivated rice were obtained from the China National Center for Bioinformation (CNCB; https://ngdc.cncb.ac.cn/), the Ensembl Plants (https://plants.ensembl.org/), the National Center for Biotechnology Information (NCBI; https://www.ncbi.nlm.nih.gov), and the Rice Genome Annotation Project (RGAP; https://rice.uga.edu) (Supplementary Table S1). PPO candidates were identified using the tBLASTn program with Nipponbare PPO sequences as queries. Genomic regions corresponding to candidate loci were retrieved and subjected to protein and coding sequence (CDS) prediction using FGENESH+ program (http://www.softberry.com), employing the Hidden Markov Model (HMM) gene model and using the protein sequences of Nipponbare PPO1 and PPO2 as references. Following the algorithm-based prediction, the splice donor site of the intron 2 of *PPO1* was manually corrected due to its atypical 5’ splice donor sequence, which contains GC dinucleotide instead of the canonical GT. Sequences of truncated PPOs were further refined by manual curation based on pairwise alignment with intact PPO homologs.

### Multiple sequence alignment and mutation sites identification of truncated *PPOs*

Multiple sequence alignments of CDS and protein sequences were performed using MUSCLE algorithm (Edgar 2022), and the results were visualized with Seaview program (Gouy et al. 2021). To identify mutation sites in truncated *PPO*s, their protein and CDS sequences were aligned to the full-length *PPO*s of closest related species, enabling detection of insertions and deletions (indels) for gene truncation.

### Phylogenetic analysis

Phylogenetic reconstruction was conducted using the PHYLIP package (Retief 2000). Firstly, protein sequences were aligned with MAFFT program, and the resulting alignments were converted into PHYLIP format. Protein distance matrices were then generated using the Protdist algorithm in PHYLIP package, and phylogenetic trees were inferred with the Neighbor-Joining (NJ) method. Bootstrap datasets were generated using Seqboot algorithm, and bootstrap supporting values were calculated with the online program BOOSTER (https://booster.pasteur.fr/new/). Final phylogenetic trees were visualized with iTOL (https://itol.embl.de/).

### Phenol color reaction assay

All rice grains used in this assay were obtained from laboratory collections or provided by Dr. Yue-Ie Caroline Hsing (Institute of Plant and Microbial Biology, Academia Sinica, Taiwan). The phenol color reaction test was conducted following the procedure of Yu et al. (2008), with minor modifications. Briefly, two replicate batches of 25 mature grains per species or lines were firstly washed thoroughly with tap water and then with distilled water to remove surface contaminants. Cleaned grains were blotted dry on paper towels and incubated in 5 mL of 3% (v/v) phenol solution at 25 °C in darkness for seven days. After incubation, grains were rinsed three times with distilled water to remove residual phenol, oven-dried overnight at 65 °C, and stored in a desiccator prior to imaging.

### RNA-seq expression analysis

Expression levels of *PPO* genes in Nipponbare and 9311 cultivars were retrieved from the Rice Annotation Project Database (RAP-DB, https://rapdb.dna.affrc.go.jp) and the Plant Public RNA-seq Database (PlantRNADb, https://plantrnadb.com), respectively. For *O. minuta*, raw RNA-seq data were downloaded from NCBI SRA archive (https://www.ncbi.nlm.nih.gov/sra) and retrieved using the fastq-dump algorithm of SRA-Toolkit (https://hpc.nih.gov/apps/sratoolkit.html). The reads were aligned to the reference genome with HISAT2 (Kim et al. 2019), and the resulting alignments were sorted and indexed using SAMtools (Li et al. 2009). Transcript assembly and quantification were performed using StringTie (Pertea et al. 2015). Expression profiles were visualized with the heatmap package in R language (Kolde 2025).

## Results

### The Nipponbare genome encodes two full-length polyphenol oxidases (PPOs) and three truncated variants

**Fig. 1:**
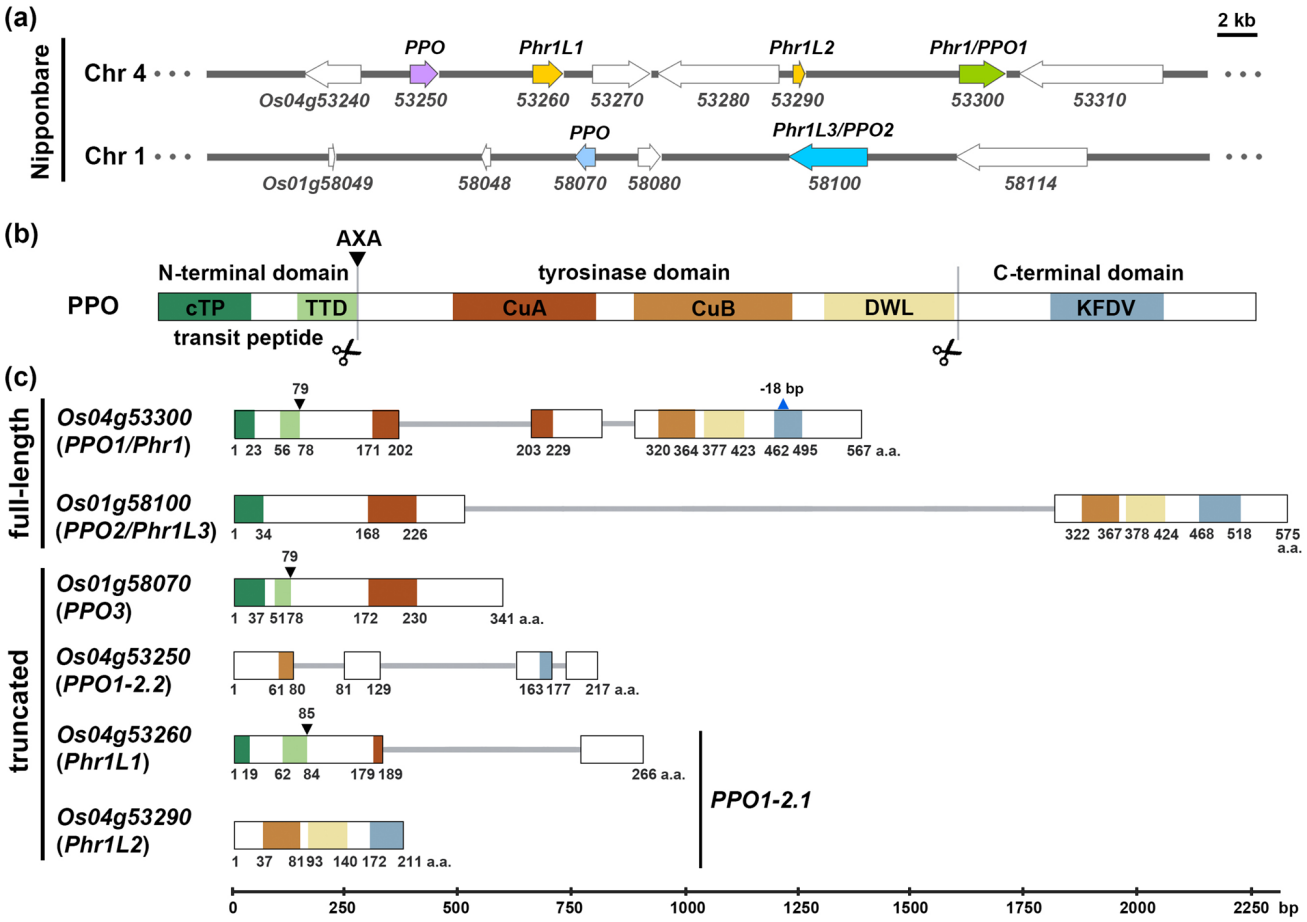
Genomic organization and structural features of *PPO* genes in *Oryza sativa* ssp. *japonica* cv. Nipponbare. Six *PPO* loci identified in the Nipponbare reference genome: *Os04g53250*, *Os04g53260* (*Phr1L1*), *Os04g53290* (*Phr1L2*), *Os04g53300* (*PPO1*/*Phr1*), *Os01g58070*, and *Os01g58100* (*PPO2*/*Phr1L3*), organized into two gene clusters on chromosomes 1 and 4. (**a**) Genomic organization of *PPO* gene clusters on chromosome 1 and 4. Colored arrows represent *PPO* genes and white arrows indicate neighboring genes. Scale bar represents 2 kb. (**b**) Protein domain architecture of a canonical PPO. Three major domains are shown: N-terminal domain with chloroplast transit peptide (cTP) and thylakoid transfer domain (TTD); tyrosinase domain with two conserved copper-binding sites (CuA and CuB) and DWL (asp-trp-leu) motif; C-terminal domain with KFDV (lys-phe-asp-val) motif. Scissor symbols mark the two cleavage sites of PPO. (**c**) Gene structures of full-length and truncated *PPO* genes. Colored boxes represent the motifs described in (**b**), with positions indicated in amino acids (a.A.). Inverted triangles mark the AXA cleavage site. *PPO1* and *PPO2* show intact gene structures with specific variations. The blue triangle denotes the 18-bp deletion in *PPO1*

Previous studies have shown that the rice genome (*Oryza sativa* ssp. *japonica* cv. Nipponbare) contains two full-length polyphenol oxidase (*PPO*) genes, *Phr1* (*Os04g53300*, *OsPPO1*) and *Phr1L3* (*Os01g58100*, *OsPPO2*), as well as two truncated paralogs on chromosome 4, *Phr1L1* (*Os04g53260*) and *Phr1L2* (*Os04g53290*), which were proposed to be pseudogenes (Yu et al. 2008). In addition, Nipponbare *PPO1* itself carries an 18 base pair (bp) in-frame deletion within the KFDV motif of the CTD, rendering it non-functional. However, the latest annotation of the reference genome (RGAP7) revealed two additional *PPO* genes: *Os01g58070* and *Os04g53250* (Fig. 1a), both of which are truncated PPO. The two full-length PPO genes, *OsPPO1* (*Os04g53300*, encoding a 570 a.a. protein) and *OsPPO2* (*Os01g58100*, encoding a 575 a.a. protein), exhibit the canonical PPO architecture: An N-terminal chloroplast transit peptide (cTP), a central tyrosinase domain containing two conserved copper-binding sites (CuA and CuB), and a C-terminal domain (CTD) that shields the active site (Figs. 1b, c). By contrast, the remaining four *PPO* genes encode truncated proteins that lack one or more conserved domains. *Os01g58070* is intronless, and its encoded 341 a.a. product retains only the N-terminal cTP and a partial tyrosinase domain containing the CuA motif. *Os04g53250* contains three introns, but its 217 a.a. product preserves only an incomplete CuB motif and part of the CTD. *Os04g53260* (268 a.a.) has a single intron and encodes the N-terminal region and a partial CuA motif followed by an uncharacterized extension. *Os04g53290* (211 a.a.) is intronless and encodes only the CuB motif together with the CTD. Our analysis revealed that *Os04g53260* and *Os04g53290* likely represent a single disrupted gene locus interrupted by two transposable elements (*Os04g53270* and *Os04g53280*). Supporting evidence for this interpretation is presented in the following sections. Collectively, these findings indicate that the Nipponbare genome contains only two full-length *PPO* genes, whereas the remaining three loci correspond to truncated or deletion-derived variants. The presence of multiple truncated *PPOs* suggests that both gene expansion and gene loss have been instrumental in shaping the *PPO* family in rice. To further elucidate the evolutionary status of these *PPO* loci and assess whether similar patterns occur across the genus, we extended our analysis to other *Oryza* species.

### The genus *Oryza* contains three distinct PPO types organized into two genomic clusters

To examine the copy number and chromosomal distribution of *PPO* genes, we analyzed 19 wild *Oryza* species with fully sequenced genomes, along with two cultivated rice species (*O. sativa* and *O. glaberrima*) and three closely related Poaceae members—*Leersia perrieri*, *Hordeum vulgare*, and *Brachypodium distachyon* (Table 1 and Supplementary Table S1). Although the genome of *O. longistaminata* has been sequenced (Li et al. 2020), it was excluded from our analysis due to incomplete coverage of the *PPO* loci. Among the investigated *Oryza* species, we found that diploids harbor three to five *PPO* genes, including both intact full-length and truncated variants, whereas the eight tetraploid species possess seven to eleven (Table 1). Notably, truncated *PPO* copies were identified in nearly all species, except *O. meridionalis* and *O. brachyantha*, as well as in the two related Poaceae species (*H. vulgare* and *B. distachyon*). Across the examined genomes, the number of truncated *PPO* genes ranged from one to eight, with *O. alta* possessing the highest copy number (Table 1).

**Table 1:** Summary of full-length and truncated PPO genes in *Oryza* species. PPOs were identified from 21 *Oryza* species and three close relatives (*Leersia perrieri*, *Hordeum vulgare*, and *brachypodium distachyon*). Truncated PPOs are defined as genes containing premature stop codons or large deletions caused by indels and frameshift mutations. The abbreviations “FL” and “Trunc” represent full-length and truncated PPOs, respectively. The “Di” and “Tetra” represent diploidy and tetraploidy, respectively

Species (genome type)	Ploidy	PPO													
		PPO1				PPO2			PPO3			Total			
		PPO1-1		PPO1-2											
		FL	Trunc	FL	Trunc	FL	Trunc		FL		Trunc	FL		Trunc	Total
**Oryza species**															
*O. sativa japonica* Nipponbare (AA)	Di	1^a^	0	0	2	1	0	0		1			2	3	5
*O. rufipogon* (AA)	Di	1	0	0	2	1	0	1		0			3	2	5
*O. sativa indica* 9311 (AA)	Di	1	0	0	1	0	1	1		0			2	2	4
*O. nivara* (AA)	Di	1	0	0	2	0	1	1		0			2	3	5
*O. barthii* (AA)	Di	1	0	0	2	0	1	1		0			2	3	5
*O. glaberrima* (AA)	Di	0	1	0	2	0	1	1		0			1	4	5
*O. meridionalis* (AA)	Di	1	0	0	0	1	0	1		0			3	0	3
*O. glumipatula* (AA)	Di	1	0	0	2	0	1	1		0			2	3	5
*O. punctata* (BB)	Di	1	0	1	1	1	0	1		0			4	1	5
*O. malampuzhaensis* (BBCC)	Tetra	1	1	0	3	2	0	1		1			4	5	9
*O. minuta* (BBCC)	Tetra	2	0	1	2	3	0	2		0			8	2	10
*O. officinalis* (CC)	Di	1	0	0	1	2	0	1		0			4	1	5
*O. alta* (CCDD)	Tetra	2	1	0	3	1	3	0		1			3	8	11
*O. latifolia* (CCDD)	Tetra	2	1	0	3	2	1	2		0			6	5	11
*O. australiensis* (EE)	Di	2	0	0	0	0	0	0		1			2	1	3
*O. meyeriana* (KK)	Di	1	0	0	0	0	2	0		1			1	3	4
*O. coarctata* (KKLL)^b^	Tetra	1	0	0	0	2	2	0		1			4^b^	3	7^b^
*O. schlechteri* (HHKK)	Tetra	1	0	0	1	2	4	0		1			3	6	9
*O. rideyi* (HHJJ)	Tetra	2	0	0	0	4	3	0		2			6	5	11
*O. longiglumis* (HHJJ)	Tetra	2	0	0	0	3	2	0		2			5	4	9
*O. brachyantha* (FF)	Di	1	0	0	0	2	0	0		0			3	0	3
*O. granulata* (GG)	Di	1	0	0	0	0	3	0		0			1	3	4
**Close relative**															
*L. perrieri*	Di	1	0	0	0	0	2	0		0			1	2	3
*H. vulgare*	Di	2	0	0	0	2	0	0		0			4	0	4
*B. distachyon*	Di	1	0	0	0	2	0	1		0			4	0	4

^a^ The *PPO1* of Nipponbare is a full-length *PPO* with 18 bp deletion without frameshift

^b^ *O. caractata* contains one unclassified *PPO*

**Fig. 2:**
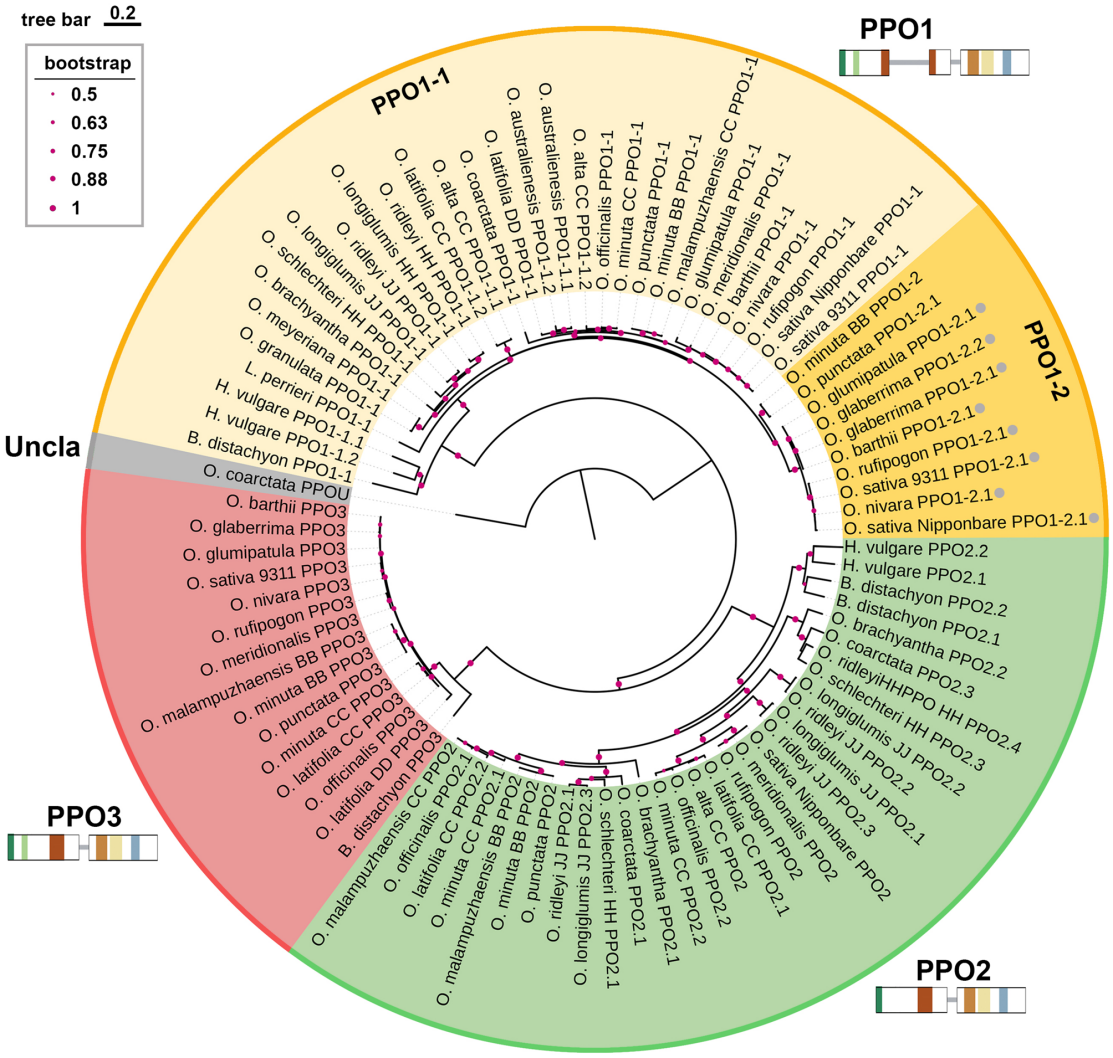
Phylogenetic relationships of PPO family proteins across *Oryza* species and related Poaceae. A phylogenetic tree of all identified full-length PPO orthologs, together with eight truncated PPO1-2 sequences from 21 *Oryza* species and three closely related Poaceae taxa (*Leersia perrieri*, *Hordeum vulgare*, and *Brachypodium distachyon*), was constructed using Neighbor-Joining (NJ) method with 1000 bootstrap replicates. Red dots mark nodes with bootstrap support greater than 0.5 with the size proportional to bootstrap value. Scale bar represents amino acid substitutions per site. Gray dots appeared in PPO1-2 group indicate the truncated sequences. “Uncla” represents the unclassified PPO of *O. coarctata.* structural features of *PPO1–3* are shown alongside the phylogenetic tree. Boxes and gray lines represent exons and introns, respectively, and conserved motifs are highlighted in color as Fig. 1b

**Fig. 3:**
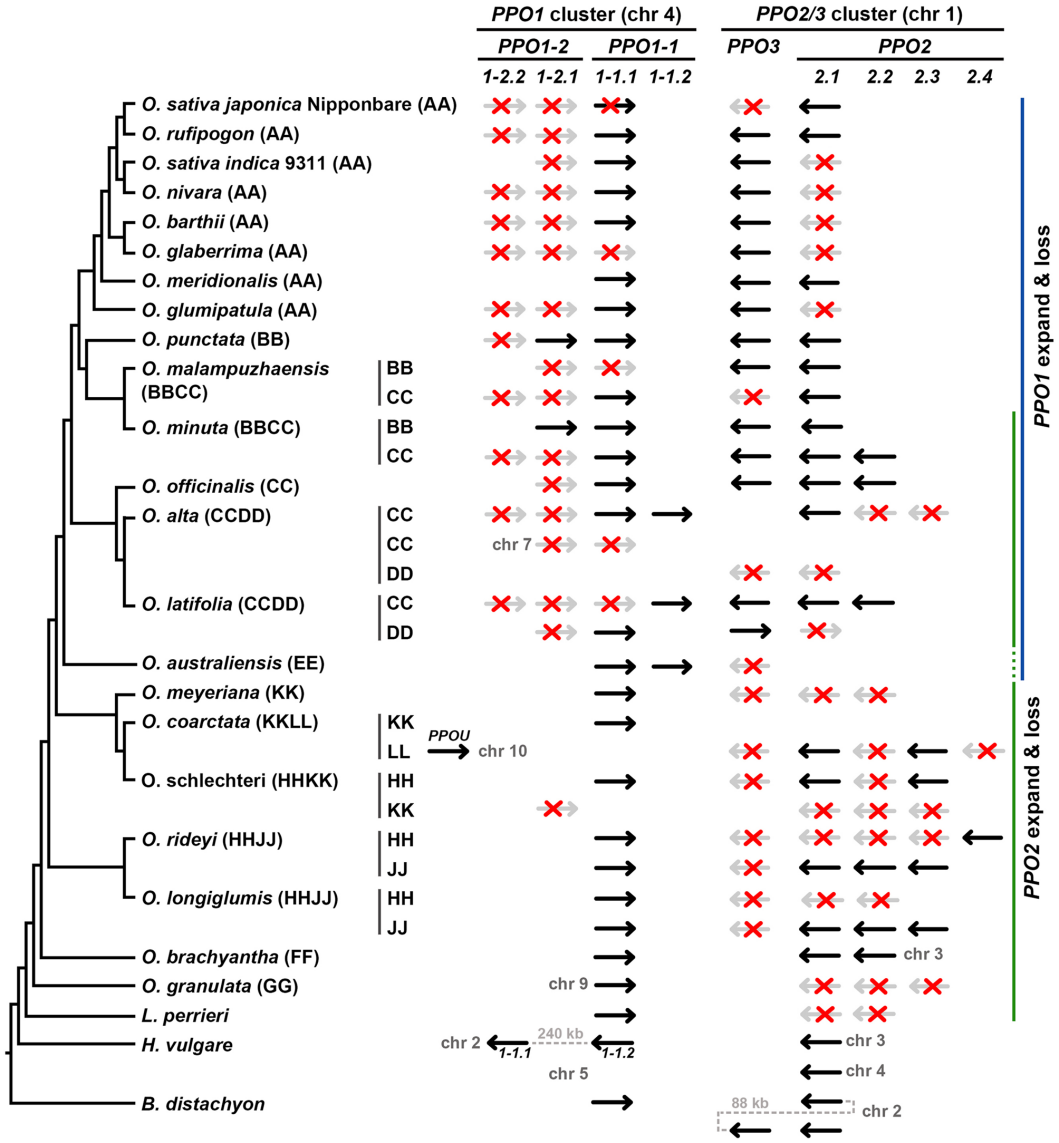
Occurrence and distribution of *PPO* genes across *Oryza* species and related Poaceae. The *PPO* gene inventory is shown for 21 *Oryza* species and three closely related Poaceae species (*Leersia perrieri*, *Hordeum vulgare*, and *brachypodium distachyon*). Species are arranged by evolutionary relationships (Fornasiero et al. 2025), with genome types indicated in parentheses (AA, BB, CC, BBCC, CCDD, etc.). The *PPO1* cluster (chromosome 4) and *PPO2/PPO3* cluster (chromosome 1) are shown separately. Black arrows denote full-length *PPO* loci; gray arrows represent truncated *PPO* loci containing premature stop codons or large deletions; and red crosses indicate nonfunctional *PPO* loci or those lacking essential domains. Numbers beside the arrows indicate the chromosomal positions of *PPO* genes located outside the canonical clusters on chromosomes 1 and 4. *PPOU* represents the unclassified PPO

To categorize the identified PPO sequences, we initially conducted phylogenetic and protein-feature analyses using primarily full-length PPO sequences (Supplementary Table S2), as most truncated PPO loci contain multiple indels that introduce frameshift and premature stop codons, resulting in incomplete proteins lacking the CTD or other essential domains. Phylogenetic analysis of the putative *PPO* protein sequences resolved three well-supported clades, designated PPO1–PPO3, with PPO1 further divided into two subtypes, PPO1-1 and PPO1-2 (Fig. 2). Although PPO1-1 and PPO1-2 share high protein sequence similarity (~88%), they were considered distinct subgroups owing to their divergent expression patterns (Supplementary Fig. S1). *PPO1-1* is primarily expressed in the palea, lemma, and endosperm, whereas transcripts of *PPO1-2* are detected predominantly in the roots, albeit at low expression levels. In contrast, *PPO2* is predominantly expressed in the leaf sheath, whereas *PPO3* transcripts are barely detectable across tested tissues. Accordingly, we designated each *PPO* gene based on its species, phylogenetic clade, and chromosomal position. In Nipponbare, the two full-length *PPO* genes—*Os04g53300* (*OsNip_PPO1-1*) and *Os01g58100* (*OsNip_PPO2*)—were assigned as Types PPO1-1 and PPO2, respectively. Using these full-length PPOs as references, we classified the truncated *PPO* loci accordingly: *Os04g53250* (*OsNip_PPO1-2.2*) and *Os04g53260/Os04g53290* (*OsNip_PPO1-2.1*) were assigned to Type PPO1-2, while *Os01g58070* (*OsNip_PPO3*) was assigned to Type PPO3 (Fig. 3). This classification system enabled us to systematically categorize all *PPO* loci across the analyzed *Oryza* species. Intriguingly, an intact PPO identified in *O. coarctata* (residing on chromosome 10 of LL genome) could not be classified into any PPO types (Figs. 2 and 3).

Genomic mapping revealed that *PPO1* genes form a cluster on chromosome 4, whereas *PPO2* and *PPO3* genes are co-localized on chromosome 1 (Figs. 1a and 3). Although intron sequences and lengths vary among *PPOs*, the number and positions of introns are highly conserved. *PPO1* contains two introns: one located within the CuA motif and another positioned upstream of the CuB motif. In contrast, both *PPO2* and *PPO3* contain only a single intron upstream of the CuB region (Fig. 2). Notably, only seven *Oryza* species possessing all three full-length PPOs, with *O. rufipogon* (AA genome), *O. meridionalis* (AA genome), *O. punctata* (BB genome), and *O. officinalis* (CC genome) being the four diploid species. Among the three closely related Poaceae species examined, only *B. distachyon* contains full-length *PPO1– PPO3*.

### PPO1 and PPO2, but not PPO3, expanded in *Oryza* through tandem duplication

Genes can expand through tandem duplication, segmental duplication, or whole-genome duplication (Birchler and Yang 2022). In our analysis, the three closely related Poaceae species contain only 3–4 *PPO* genes, whereas *Oryza* species harbor up to 11 copies (Table 1). Notably, all duplicated *PPO* genes are organized into either *PPO1* or *PPO2/PPO3* clusters adjacent to their progenitor genes, indicating that *PPO* expansion in *Oryza* occurred predominantly through tandem duplication (Fig. 3). Specifically, *PPO1* expanded in species with AA, BB, CC, DD or EE genomes, whereas *PPO2* expanded in species with CC, JJ, KK, LL, FF, GG, and HH genomes. Consequently, CC genome is the only one exhibiting duplications of both *PPO1* and *PPO2*. Notably, no *PPO3* duplications were observed in any species examined. Among the three closely related Poaceae species, *H. vulgare* contains one duplicated *PPO1*, while *L. perrieri* and *B. distachyon* each contain one duplicated *PPO2*; however, both *PPO2* copies in *L. perrieri* are truncated (Fig. 3). Although *PPO* duplications also occur in these Poaceae relatives, their copy numbers remain substantially lower than those observed in *Oryza* species.

### PPO2 lacks the thylakoid transfer domain

**Fig. 4:**
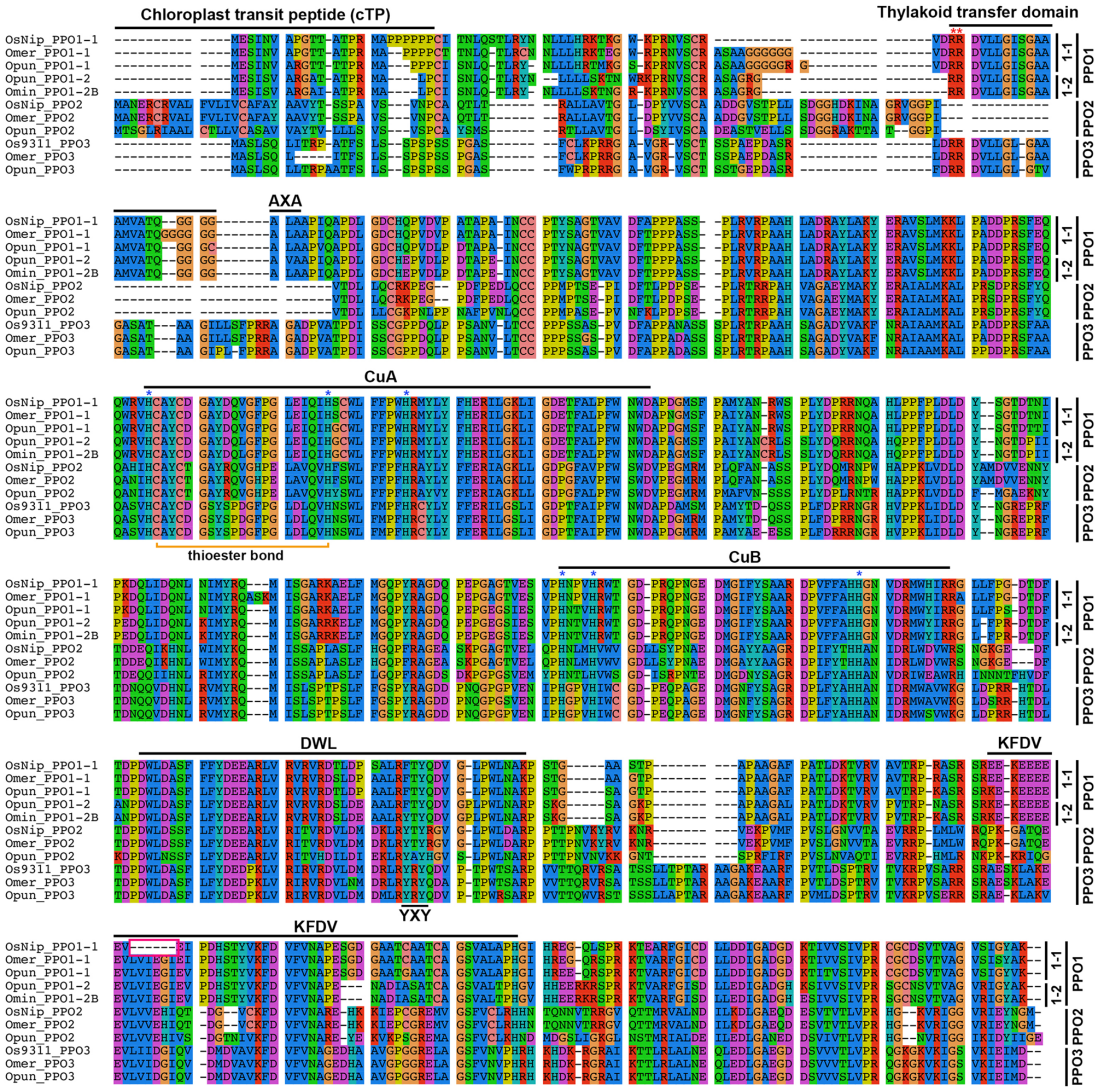
Multiple sequence alignment of three *Oryza* PPO types. The PPO1–3 protein sequences from four *Oryza* species—*O. sativa* ssp. *japonica* Nipponbare (OsNip), *O. meridionalis* (omer), *O. punctata* (opun), and *O. minuta* (omin)—were aligned using MUSCLE with subsequent manual refinement. Conserved functional motifs are annotated as follows: CuA and CuB represent the two copper-binding sites within the central tyrosinase domain; the cysteine–histidine thioester bond located in CuA is marked in yellow. Blue asterisks denote conserved histidine residues in the CuA (three residues) and CuB (three residues) copper-binding motifs, while the red asterisk indicates the twin-arginine motif for Tat pathway signal recognition. AXA and YXY represent conserved alanine cleavage and tyrosine motifs, respectively. The 18 bp deletion (corresponding to six amino acid residues) in Nipponbare PPO1 is highlighted with a magenta open box. Additional conserved motifs include the DWL and KFDV motifs following the tyrosinase domain

Plant PPOs belong to the type-3 copper oxidase family and contain a binuclear copper active site (CuA and CuB), each coordinated by three conserved histidine residues (Kaintz et al. 2014). Most plant PPOs are synthesized and transported to plastids as latent precursors and become activated only after proteolytic removal of the C-terminal domain containing KFDV motif (Fuerst et al. 2014). Multiple sequence alignment revealed that both CuA and CuB are highly conserved across all three PPO types. In addition, the DWL and KFDV motifs within the CTD are also strongly conserved (Fig. 4). By contrast, the tyrosine motif (YXY) shows a slight variation in *PPO1*, where it appears as FTY. The strong conservation of these motifs and the CTD suggests that all three *PPO* types in rice may retain similar enzymatic properties and substrate specificity. Despite this sequence conservation, PPO2 exhibits a divergent N-terminal domain (NTD) compared to PPO1 and PPO3 (Fig. 4). Most plant PPOs are thylakoid lumen proteins transported into chloroplasts via either the Tat (twin-arginine-translocation) or Sec-dependent pathways. Sequence analysis revealed that the NTDs of PPO1 and PPO3 contain both a cTP and a thylakoid transfer domain (TTD), whereas PPO2s carries only a cTP and lack the entire TTD. Consequently, the characteristic twin-arginine (RR) and alanine cleavage (AXA) motifs required for Tat pathway targeting are present in PPO1 and PPO3 but absent in PPO2.

### *PPO1-2* and *PPO2* each show conserved mutation sites across species, whereas *PPO1-1* and *PPO3* exhibit independent mutations

**Fig. 5:**
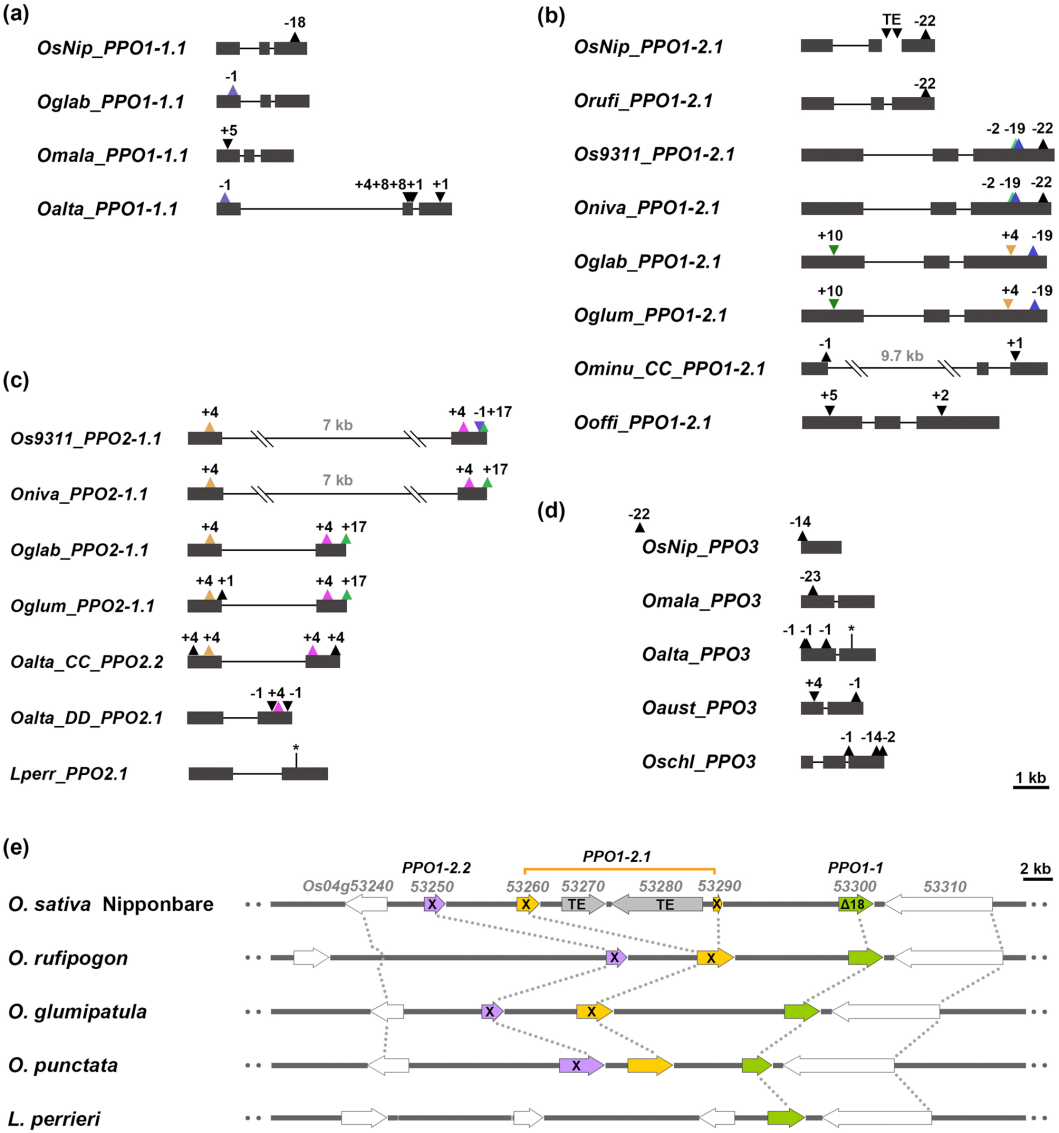
Conserved and independent mutations of *PPO* gene alleles resulting in nonfunctional and truncated proteins. (**a**) *PPO1-1* mutations, (**b**) *PPO1-2* mutations, (**c**) *PPO2* mutations, and (**d**) *PPO3* mutations showing indels within *PPO* gene exons. Upward triangles represent base pair insertions, while inverted triangles indicate deletions. Asterisks denote point mutations resulting in premature termination codons. “TE” indicates transposable element insertions. Gene nomenclature follows the pattern: species name, genome type, and chromosomal location as presented in Fig. 3. (**e**) Synteny analysis of the *PPO1* gene cluster across selected species. Colored arrows represent *PPO* genes and white arrows indicate neighboring genes. Truncated *PPO* genes are marked with an “X”, while full-length *PPO* genes containing an 18 bp deletion are denoted as “Δ18”. Syntenic gene relationship are connected by dashed lines

Most truncated *PPO* genes contain indels that introduce premature stop codons and truncated functional domain. Previous studies reported that the loss-of-function phenotype of *PPO1-1* in *O. sativa* ssp. *japonica* cv. Nipponbare—a genotype prevalent across Asia—resulted from an 18-bp in-frame deletion that arose during domestication (Yu et al. 2008). However, the evolutionary patterns of mutations in other *PPO* types, particularly those occurring in wild *Oryza* species, remain unknown. To characterize these mutation patterns, we mapped the types and locations of indels in representative truncated *PPO* genes (Fig. 5). Our analysis revealed two distinct patterns: mutation sites in *PPO1-1* and *PPO3* arose independently in different species, whereas those in *PPO1-2* and *PPO2* are conserved across species (Fig. 5a–d). Notably, *PPO1-2* and *PPO2* each contain 3–4 mutation sites per gene, which is higher than the number observed in *PPO1-1* and *PPO3*. The conserved mutations in *PPO2* and *PPO1-2* exhibit genome-specific distribution patterns. In *PPO2*, two 4 bp insertion sites (respectively located in exon 1 and 2) are present in species with AA or CC genomes, while a 17 bp insertion site in exon 2 occurs exclusively in AA genome species (Fig. 5C). Similarly, *PPO1-2* mutations are conserved only within AA genome species (Fig. 5B): a 19 bp deletion is shared among *O. glumipatula*, *O. glaberrima*, *O. nivara*, and *O. sativa* ssp. *indica* cv. 9311, whereas a 22 bp deletion is present in *O. nivara*, *O. rufipogon*, *O. sativa* ssp. *japonica* Nipponbare, and *O. sativa* ssp. *indica* cv. 9311. In contrast, no conserved mutation sites were observed in *PPO1-2* among species with non-AA genomes.

### The Nipponbare *PPO1-2.1* (*Os04g53260*/*Os04g53290*) represents a single gene disrupted by two transposons

Transposon elements (TE)-related sequences account for 40% of the rice genome and have played key roles in rice evolution and domestication (Jiang et al. 2013). In Nipponbare, the two truncated loci *Os04g53260* and *Os04g53290*, which encode the *N*- and C- terminal portions of a putative full-length *PPO*, are interrupted by two Ty3-gypsy transposons (Fig. 5e). We therefore hypothesized that these two *PPO* loci originated from a single ancestral *PPO* gene, with *Os04g53260* corresponding to the N-terminal region (including N-terminal transition peptide and partial CuA motif) and *Os04g53290* corresponding to the C-terminal (including CuB, DWL and KFDV motifs), respectively. To test this hypothesis, we compared the genomic region containing the *PPO1* cluster in Nipponbare with those of three *Oryza* species (*O. rufipogon, O. glumipatula* and *O. punctata*) and *L. perrieri*. The *PPO1* cluster was found to be syntenic across all species examined. All three *Oryza* species contain a *PPO1-2.1* locus at the corresponding position without transposon insertions, supporting the conclusion that the two truncated *PPO* loci in Nipponbare, *Os04g53260* and *Os04g53290*, resulted from TE-mediated gene disruption (Fig. 5e). Notably, *O. rufipogon* and *O. glumipatula* also carry truncated *PPO1-2.1* with mutation patterns in *O. rufipogon* closely resembling those in Nipponbare (Fig. 5b). Expression analysis further revealed that transcripts of *Os04g53260* were weakly detectable in roots, whereas *Os04g53290* showed no expression. This suggests that only *Os04g53260*, the N-terminal fragment, retains the original promoter region (Supplementary Fig. S1).

### A novel truncated allele of *PPO1-1* was identified in the cultivated rice *Oryza glaberrima*

**Fig. 6:**
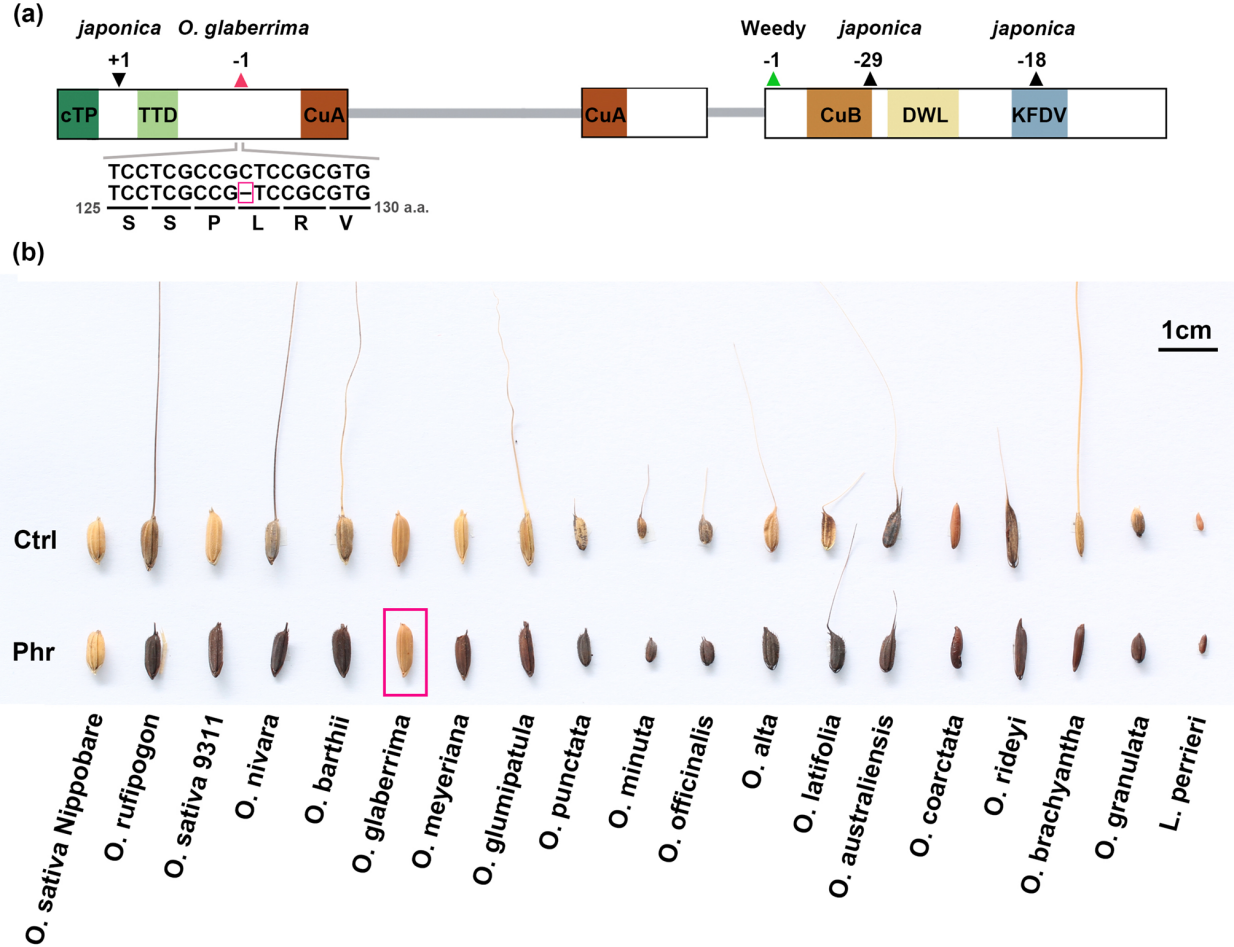
A novel *PPO1-1* mutant allele in *Oryza glaberrima* and phenol color reaction assay. (**a**) Identification of a new *PPO1-1* mutant allele in *O. glaberrima*. Previously reported mutation sites in *O. sativa* spp. *japonica* (black triangles) and weedy rice (green triangles) are shown (Yu et al. 2008; Gross et al. 2009), along with a novel single-base deletion at amino acid position 128 identified in *O. glaberrima* (red triangle). (**b**) Phenol color reaction assay for PPO activity. Grains from *O. glaberrima* (IRGC: 96,717), two *O. sativa* cultivars (ssp. *japonica* cv. Nipponbare and ssp. *indica* cv. 9311), 15 wild *Oryza* species, and *Leersia perrieri* were tested. All grains retained the lemma and palea, except for *O. coarctata*, which was peeled to reveal the positive phenol reaction of the inner seed, as the hull initially showed a negative reaction. “Ctrl” and “phr” denote grains without and with phenol incubation, respectively. Scale bar = 1 cm

Mutations in *PPO1-1* loci have been reported primarily in domesticated rice, including *O. sativa* cv. Nipponbare and weedy rice (Yu et al. 2008; Gross et al. 2009). In our analysis, we identified a truncated *PPO1-1* allele in the cultivated African rice *O. glaberrima*, caused by a novel single-base deletion in exon I (Fig. 6a). To determine whether this mutation is specific to the reference *O. glaberrima* genome (IRGC accession: 96717) or more widespread, we examined seven additional *O. glaberrima* cultivar genomes and found that three also carried this allele (Supplementary Table S3). Consistent with a loss-of-function mutation phenotype, phenol color reaction assay for PPO activity of the reference *O. glaberrima* accession (IRGC 96717) yielded a negative result (Fig. 6b).

## Discussion

Previous studies on rice polyphenol oxidases (PPOs) have primarily focused on *Phr1* (*PPO1*), identifying loss-of-function alleles in *japonica* cultivars and characterizing its role in the grain phenol reaction phenotype (Yu et al. 2008; Gross et al. 2009). However, the complete composition of the *PPO* gene family, its structural diversity, and evolutionary dynamics across the *Oryza* genus have remained largely unexplored. In this study, we systematically surveyed *PPO* genes across 21 *Oryza* species representing diverse genome types and their close relatives within the Poaceae, revealing three distinct PPO types (PPO1–PPO3) and a widespread occurrence of both full-length and truncated variants. Among these, PPO3 represents a previously undescribed group: in *Oryza sativa* ssp. *japonica* cv. Nipponbare, it corresponds to *Os01g58070*, which is massively truncated. Our results also indicate that all three PPO types are conserved in close relatives of *Oryza* in the Poaceae family, suggesting their existence in the common ancestor of *Oryza* and related grasses.

The copy number of *PPOs* in the *Oryza* genus is highly dynamic, with gene duplication further shaping this diversity. Specifically, *PPO1* and *PPO2* have undergone tandem duplications in a majority of *Oryza* species, and these events correlate strongly with phylogenetic relationships and genome type classification. *Oryza* species are conventionally grouped into three gene pools: the primary gene pool (GP1, *O. sativa* complex, AA genome), the secondary gene pool (GP2, *O. officinalis* complex, BB–EE genome types), and the tertiary gene pool (GP3, *O. ridleyi* complex and others, FF–LL genome types) (Solis et al. 2020). Based on PPO copy number patterns, the examined *Oryza* species can be broadly classified into three corresponding *PPO* expansion groups (Fig. 3 sidebar): Group I includes the most derived species with only *PPO1* expansion (AA and BB genomes), corresponding to GP1 and certain diploid and tetraploid BB genome species; Group II includes intermediate species with expansions in both *PPO1* and *PPO2* (CC–DD genomes), largely corresponding to GP2; and Group III includes more ancestral lineages featuring only *PPO2* expansion (FF–LL genomes), corresponding to GP3. The phylogenetic distribution of these expansion patterns also reveals a dynamic trajectory of gene family evolution across the *Oryza* genus, consistent with the established *Oryza* phylogeny (Fornasiero et al. 2025). Notably, basal or early-diverging *Oryza* lineages (FF–LL genomes) exhibit extensive *PPO2* copies but lack *PPO1* duplications, whereas the most recently diverged lineages with AA and BB genomes—including cultivated rice and its wild relatives—show the opposite pattern. They have lost *PPO2* expansions while retaining or further expanding *PPO1*. Species with CC and DD genomes, which occupy an intermediate phylogenetic position, retain both *PPO1* and *PPO2* expansions. These complementary distribution patterns strongly support a sequential duplication model: *PPO2* expansion occurred early in *Oryza* evolution, followed by *PPO1* duplication in more derived lineages, with a subsequent selective loss of duplicated *PPO2* genes in the AA–BB ancestor. The coexistence of both expansions in CC–DD species thus represents a transitional evolutionary state.

The observed patterns of *PPO* expansion and loss likely reflect adaptive responses to shifting environmental pressures during *Oryza* evolution. Given that PPOs are widely implicated in plant defense against herbivores and pathogens through phenolic oxidation (Thipyapong et al. 2007; Zhang 2023), we propose that these expansion events might be correlated with specific instances of heightened selective pressure. Our analysis suggests that the expansion of *PPO2* arose in the common ancestor of the genus *Oryza* and the related grass *Leersia perrieri* but was subsequently lost in GP1 and BB genome lineages. This ancestral expansion may reflect an adaptation to high pathogen pressure on vegetative tissues (aerial parts), strongly corresponding to the shaded forest habitats typically occupied by GP3 species (Vaughan et al. 2003; Solis et al. 2020). In contrast, the subsequent expansion of *PPO1* in the ancestor of GP1 and GP2 species suggests a shift in selective pressure toward reproductive tissues (seeds), potentially driven by the transition to open wetland habitats characteristic of these more derived lineages. While the correlation between these genomic patterns and current habitat preferences—shaded forests for GP3 versus open wetlands for GP1 and GP2—is distinct, present-day habitat distributions may not accurately mirror ancestral environments. Therefore, these interpretations remain hypothesis-generating, and additional comparative ecological, transcriptomic, and functional studies will be required to establish causal links between PPO diversification and environmental adaptation.

Although the *PPO* gene family expanded in *Oryza* species, most genomes retain fewer than five full-length copies, reflecting frequent gene loss. This pattern suggests that maintaining multiple functional *PPO* copies may not be advantageous or may even be deleterious. Yet several studies have shown that PPO overexpression enhances plant defense against pathogens and herbivores in species such as strawberry, poplar and tomato (Li and Steffens 2002; Wang and Constabel 2004; Jia et al. 2016). However, in walnut, PPO overexpression caused cell death, and no viable transgenic lines could be obtained (Araji et al. 2014), suggesting that excessive PPO activity can be harmful. In rice, although comparable overexpression data are unavailable, the frequent loss of PPO genes following expansion may reflect a balance between retaining defensive functions and avoiding potential costs such as accelerated grain browning or altered phenolic metabolism. Interestingly, almost all *Oryza* species retain at least one *PPO* gene in both the *PPO1* and *PPO2/PPO3* clusters, with the exception of certain cultivated rice accessions. This conservation pattern suggests that basal PPO activity remains essential for *Oryza* fitness, despite *PPO* mutants showing no obvious effects on vegetative growth aside from the absence of grain browning (Yu et al. 2008). PPO have been proposed to contribute to seed dormancy and pathogen resistance: in wild oat (*Avena fatua* L.), PPO activity in the lemma and palea increases in response to the infection by seed-decaying *Fusarium* fungi, highlighting a role in seed protection under natural conditions (Fuerst et al. 2014).

Our results fit the birth-and-death model of multigene family evolution, in which sequential duplication and lineage-specific pseudogenization events shape gene family diversity (Nei et al. 1997; Nei and Rooney 2005). In plants, many large gene families—such as resistance (R) genes, transcription factors, and enzymes involved in secondary metabolism—exhibit clear signatures of birth-and-death evolution (Michelmore and Meyers 1998; Nam et al. 2004; Barbosa et al. 2023). Recurrent tandem or segmental duplications generate new paralogs, while older copies often accumulate deleterious mutations or become pseudogenes (Sampedro et al. 2005; Wang et al. 2018). The *PPO* gene family in *Oryza* exemplifies this dynamic process: tandem duplications expanded *PPO1* and *PPO2* in lineage-specific patterns, while frequent truncations reflect ongoing pseudogenization. Truncated *PPO*s with premature termination codons display both conserved and independent mutation patterns across *Oryza* species. Loss-of-function *PPO*s has been associated with domestication, natural selection, and transposon insertion (Gross et al. 2009; Taketa et al. 2010; Inoue et al. 2015; Balarynová et al. 2022).

Our results indicated that mutations in *PPO1-1* and *PPO3* genes arose independently across different lineages (Fig. 5), suggesting that these mutations originated after speciation. The presence of *PPO1-1* mutations in both cultivated rice species—*O. sativa* ssp. *japonica* cv. Nipponbare and *O. glaberrima*—strongly supports the association of these alleles with domestication, as loss-of-function *PPO* alleles reduce enzymatic browning and improve grain appearance (Yu et al. 2008; Gross et al. 2009). By contrast, *PPO3* mutations are predominantly found in wild rice species, suggesting that natural selection, rather than domestication, drives *PPO3* loss. Notably, *PPO3* truncation shows a strong correlation with *PPO2* expansion across most *Oryza* lineages (Fig. 3), with exceptions of *O. sativa* cv Nipponbare, *O. malampuzhaensis*, and *O. australiensis*. This correlation suggests functional redundancy between *PPO2* and *PPO3*, whereby *PPO2* expansion may have reduced selective pressure to maintain *PPO3*, eventually leading to its degeneration or loss in several lineages. The truncated *PPO3* allele in Nipponbare represents an exception to this general pattern, as it is the only AA-genome species exhibiting *PPO3* loss, suggesting this may be a domestication-associated event rather than a consequence of *PPO2* expansion. Unlike truncated *PPO1-1* and *PPO3*, several mutations in *PPO1-2* and *PPO2* are conserved across species, implying that these alleles arose in common ancestors rather than during recent domestication or speciation events. Moreover, the two transposon insertions in Nipponbare *PPO1-2.1* may be related to domestication (Fig. 5B). Since both Nipponbare and *O. rufipogon* share a conserved 22 bp deletion in exon 3, the transposon insertions likely occurred after this deletion event. Previous studies reported that transposons were activated during rice domestication in Nipponbare and *O. sativa indica* cv. 9311, raising the possibility that the transposon insertions in *PPO1-2* were also domestication driven (Wang et al. 2022).

Beyond copy number variation, the three PPO types also exhibit distinct structural features. Among these, the N-terminal transient peptides composition differs significantly and likely determines subcellular localization and functional specialization (Tran and Constabel 2011). Both PPO1 and PPO3 contain a chloroplast transit peptide (cTP), a thylakoid transit domain (TTD), and twin-arginine (RR) motifs characteristic of the Tat pathway, suggesting localization to the thylakoid membrane. In contrast, PPO2 possesses only a cTP and completely lacks the TTD, indicating potential localization to the chloroplast stroma, envelope, or other plastid structures such as amyloplasts. This spatial separation may reflect distinct functional roles among the three PPO types. In addition, transcript analysis revealed that *PPO1* and *PPO2* are readily detectable, whereas PPO3 expression is very low across tested tissues. This low expression of *PPO3*, combined with its potential functional redundancy with the more accessible *PPO2*, may explain the sequential loss of *PPO3* accompanying *PPO2* gene expansion. Furthermore, phenol staining of *O. glaberrima grains*, which carry truncated *PPO1-1* but intact *PPO3*, yields a negative result, suggesting that *PPO3* may not expressed in the grain in the same manner as *PPO1-1*.

In conclusion, this study reveals that the *PPO* gene family in *Oryza* has undergone dynamic evolution shaped by sequential gene duplications, lineage-specific losses, and both natural and artificial selection. These findings provide new perspectives on PPO function, evolution, and the role of gene birth-and-death dynamics in shaping crop genomes.

## Electronic supplementary material

Below is the link to the electronic supplementary material.

Supplementary Material 1

Supplementary Material 2

## Data Availability

All analyzed or generated data is included in this article. The data analyzed or generated in this study can be obtained from the corresponding author with upon reasonable request. The data presented in this study are available on request from the corresponding author. All databases used in the study are open for public access, including the China National Center for Bioinformation (CNCB; https://ngdc.cncb.ac.cn/), the Ensembl Plants (https://plants.ensembl.org/), the National Center for Biotechnology Information (NCBI; https://www.ncbi.nlm.nih.gov), and NCBI SRA archive (https://www.ncbi.nlm.nih.gov/sra). The accession numbers of the investigated genome databases for this study are listed in Supplementary Table S1.

## Abbreviations

CDS: Coding sequence
CTD: C-terminal domain
cTP: Chloroplast transit peptide
HMM: Hidden Markov Model
Indels: Insertions and deletions
NJ: Neighbor-Joining
NTD: N-terminal domain
PPO: Polyphenol oxidase
Tat: Twin-arginine translocation
TTD: Thylakoid transit domain
R gene: Resistance gene
RR: Twin-arginine
TE: Transposon elements
