# Supplementary material for: Birth and death evolution of polyphenol oxidase (PPO) gene family in Oryza species
Source: Bot Stud. 2026 Feb 5;67:4. doi: 10.1186/s40529-026-00491-5 (PMC12876524; doi:10.1186/s40529-026-00491-5)
Supplement: Supplementary file 1 — Supplementary Material 1 [file 40529_2026_491_MOESM1_ESM.pdf]

**Supplementary Table S1. Sources and accession numbers for the species investigated in this study.** Genomic and protein sequence data were obtained from CNCB (https://ngdc.cncb.ac.cn/), Ensembl Plants (https://plants.ensembl.org/), NCBI (https://www.ncbi.nlm.nih.gov/), and RGAP (https://rice.uga.edu/).

| Species | Genome | ploidy | Database | Accession number |
|---|---|---|---|---|
| *Oryza sativa japonica* cv. Nipponbare | AA | Diploid | RGAP | GCA_001433935.1 |
| *Oryza rufipogon* | AA | Diploid | NCBI | GCA_037997075.1 |
| *Oryza sativa indica* cv. 9311 | AA | Diploid | CNCB | GWHFLAP00000000.1 |
| *Oryza nivara* | AA | Diploid | NCBI | GCA_000576065.2 |
| *Oryza barthii* | AA | Diploid | NCBI | GCA_000182155.4 |
| *Oryza glaberrima* | AA | Diploid | NCBI | GCF_000147395.1 |
| *Oryza meridionalis* | AA | Diploid | NCBI | GCA_047496205.1 |
| *Oryza glumipatula* | AA | Diploid | NCBI | GCA_000576495.2 |
| *Oryza punctata* | BB | Diploid | NCBI | GCA_000573905.2 |
| *Oryza malampuzhaensis* | BBCC | Tetraploid | NCBI | GCA_048564985.1 |
| *Oryza minuta* | BBCC | Tetraploid | NCBI | GCA_048166525.1 |
| *Oryza officinalis* | CC | Diploid | NCBI | GCA_008326285.1 |
| *Oryza alta* | CCDD | Tetraploid | NCBI | GCA_047899615.1 |
| *Oryza latifolia* | CCDD | Tetraploid | NCBI | GCA_048174585.1 |
| *Oryza australiensis* | EE | Diploid | CNCB | GWHFIGT00000000.1 |
| *Oryza meyeriana* | KK | Diploid | NCBI | GCA_047899645.1 |
| *Oryza coarctata* | KKLL | Tetraploid | NCBI | GCA_030770085.1 |
| *Oryza schlechteri* | HHKK | Tetraploid | NCBI | GCA_048320915.1 |
| *Oryza rideyi* | HHJJ | Tetraploid | NCBI | GCA_047772315.1 |
| *Oryza longiglumis* | HHJJ | Tetraploid | NCBI | GCA_048014755.1 |
| *Oryza brachyantha* | FF | Diploid | NCBI | GCA_000231095.3 |
| *Oryza granulata* | GG | Diploid | NCBI | GCA_005223365.2 |
| *Leersia perrieri* | -- | Diploid | NCBI | GCA_000325765.3 |
| *Hordeum vulgare* | -- | Diploid | Ensembl | GCA_904849725.1 |
| *Brachypodium distachyon* | -- | Diploid | NCBI | GCA_000005505.4 |

**Supplementary Table S3. *PPO1* alleles in eight *Oryza glaberrima* cultivars.** All genome assemblies were downloaded from NCBI (https://www.ncbi.nlm.nih.gov/). The assembly accession numbers and assembly names correspond to GenBank genome assembly identifiers. The IRGC accession and country of collection were searched from IRRI or web. *PPO1* alleles carrying the one–base-pair deletion are denoted as "-1," whereas those without the mutation are indicated as "WT."

| Assembly Accession | Assembly Name | IRGC Accession | Country | Genotype |
|---|---|---|---|---|
| GCA_000147395.3 | OglaRS2 | 96717 | Senegal | -1 |
| GCA_965117855.1 | Og132_Mg12_LD | 103544 | Mali | WT |
| GCA_965117915.1 | Og172_TOG7291_EH | 104589 | Burkina Faso | -1 |
| GCA_965117845.1 | Og20_TOG5438_LS | 96751 | Nigeria | WT |
| GCA_965117905.1 | TOG5681 | 96793 | Nigeria | WT |
| GCA_965117875.1 | Og182_TOG7455_HR | None | Burkina Faso | -1 |
| GCA_965117805.1 | Og103_TOG6698_LF | None | Liberia | -1 |
| GCA_965117825.1 | Og44_TOG5672_MH | None | Nigeria | WT |

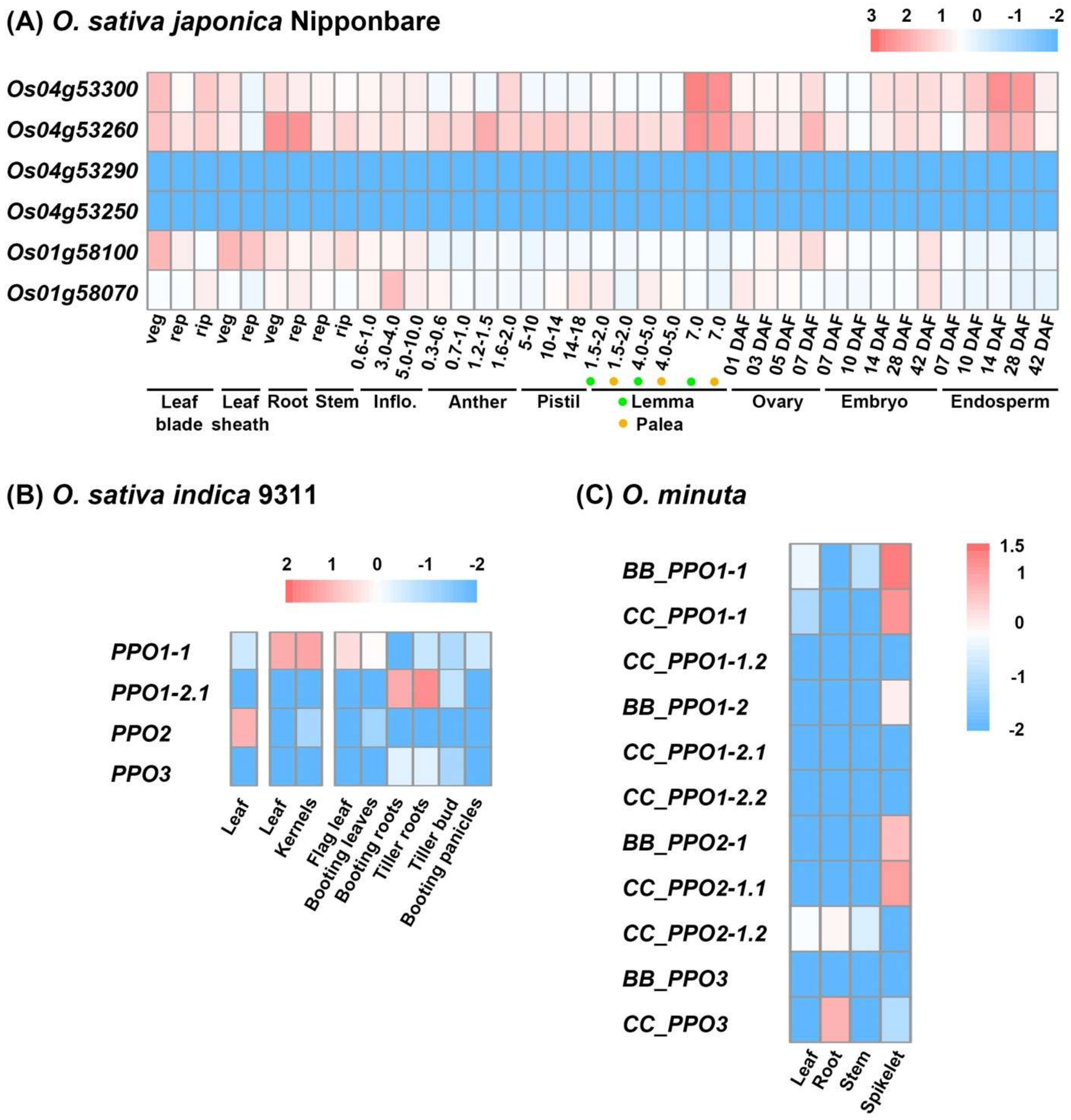


**Supplementary Figure S1. Transcript levels of PPO genes in *Oryza sativa japonica* Nipponbare, *O. sativa indica* 9311, and *O. minuta* across various tissues.** (A) Transcript levels of *O. sativa* japonica (Nipponbare) in 11 tissues. Expression data were obtained from the Rice Annotation Project Database (RAP-DB, https://rapdb.dna.affrc.go.jp). Each tissue includes two to five developmental stages with three biological replicates. Average expression values were $\log_{10}$-transformed after adding 0.01. "Inflo" indicates inflorescence; "Vet," "Rep," and "Rip" represent

vegetative, reproductive, and ripening stages, respectively. For inflorescence, anther, and lemma/palea, the unit is millimeters (mm); for pistil, the unit is centimeters (cm). DAF indicates days after fertilization. (B) Transcript levels of PPO genes in eight tissues of *O. sativa indica* 9311. Expression data were obtained from the Plant Public RNA-seq Database (PlantRNAdb, https://plantrnadb.com/). Each sample was analyzed in two biological replicates, and average values were $\log_{10}$-transformed after adding 0.01. (C) Transcript levels of PPO genes in four tissues of *O. minuta*. Raw RNA-seq data were downloaded from the NCBI SRA archive (accessions: CRR1299413–CRR1299426) and processed using the HISAT2 pipeline for alignment and expression quantification. Each tissue was analyzed in three biological replicates, and average values were $\log_{10}$-transformed after adding 0.01. Tissue names are indicated below each panel.
